# Polydimethylsiloxane Sponge-Supported Metal Nanoparticles as Reusable Catalyst for Continuous Flow Reactions

**DOI:** 10.3390/nano12122081

**Published:** 2022-06-16

**Authors:** Sergio Gómez-Graña, Marta Pita, Paula Humada-Iglesias, Jorge Pérez-Juste, Pablo Hervés

**Affiliations:** 1 CINBIO, Departamento de Química Física, Universidade de Vigo, 36310 Vigo, Spain; pitfermarta@gmail.com (M.P.); humadapaula@gmail.com (P.H.-I.); juste@uvigo.es (J.P.-J.); 2 Instituto de Investigación Sanitaria Galicia Sur, Hospital Álvaro Cunqueiro, 36213 Vigo, Spain

**Keywords:** nanomaterials, catalyst, flow reactors

## Abstract

In this manuscript, polydimethylsiloxane (PDMS) sponges supporting metal nanoparticles (gold and palladium) were developed and their catalytic properties were studied through a model reaction such as the hydrogenation of p-nitrophenol. Different synthetic conditions for gold and palladium were studied to obtain the best catalyst in terms of nanoparticle loading. The as-prepared catalysts were characterized by different techniques such as scanning electron microscopy (SEM) and inductively coupled plasma optical emission spectroscopy (ICP-OES). The catalytic efficiency and recyclability of the supported catalyst were tested in static conditions. In addition, thanks to the porous structure of the material where the catalytic centers (metal nanoparticles) are located, the model reaction for continuous flow systems was tested, passing the reaction components through the catalyst, observing a high efficiency and recyclability for these systems.

## 1. Introduction

Since Haruta [1] discovered the catalytic capacity of gold nanoparticles in 1997, a great effort has been made in the research of nanocatalysis of different types of chemical reactions [2,3,4,5,6,7]. Due to their small sizes, metal nanoparticles (NPs) have a large surface-to-volume ratio, which made them appear as excellent replacements for their bulk counterpart [8,9,10,11]. The large surface area of colloidal suspensions of metal nanoparticles provides a ready contact with the reactants, thereby improving the catalytic activity [12,13,14,15], but at the same time, the high surface energy increases their tendency to aggregate, reducing the surface-to-volume ratio, which results in the loss of catalytic activity. Then, the use of stabilizing agents is required [16], but the surfactant or capping agents needed for the colloidal stability reduce the number of active sites for the reaction. Besides, after the catalysis, the colloidal NPs are not easy to recover due to aggregation during the filtration or centrifugation processes affecting the recyclability of the system [17].

The usual approach to overcome stability and recyclability problems is anchoring the metal NPs on different kinds of support materials. Solid supports effectively inhibit the aggregation of the catalyst. The large surface area of the supporting materials and the absence of capping agents let the NPs achieve high catalytic activity [18,19,20,21,22,23]. Besides, solid supports present additional advantages, like the ease of handling and recovery from the reaction medium, facilitating the recyclability of the catalysts. There are a large number of supports for which it has been proven that metallic NPs can be an anchor for use as catalysts. Polymers [24,25] and thermoresponsive polymers [26,27] are used with no additional surface modification to protect the particles from coagulation. Silica and titanium metal oxide have also been used to immobilize metallic NPs [28,29,30,31,32] and other solid supports such as graphene [33,34,35,36], carbon nanotubes [37,38], and metal-organic frameworks [39] also achieve both high catalytic activity and reusability. Anyway, the isolation and recycling processes of these supported catalysts after each batch of reaction are tedious and wasteful because the nano- or micro dimension of the support materials making their separation by filtration or centrifugation necessary.

An innovative approach is to load NPs onto highly stable supports of macroscopic dimensions to obtain catalysts that can be handled easily as compared with the floating catalyst, addressing the challenges of reactivity, stability, and facile recyclability [23]. This type of catalyst is usually called a “dip-catalyst” because its dipping or removal from the reaction medium decides whether the reaction is catalyzed or not [24,40]. Cellulose filter paper support has been used as a dip-catalyst. The load of metal NPs can be carried out by (a) in situ reduction of metal ions on the support [41] or (b) anchoring pre-synthesized metal nanoparticles onto the support [40,41]. This last approach, used in our laboratory [42,43], offers better control of the size and shape of the nanoparticles than the in situ reduction method, but the immobilization of the NPs onto the support requires a multistep synthesis process. 

An alternative and interesting support material is polydimethylsiloxane (PDMS). PDMS is one of the most used silicon-based organic polymers due to its high versatility, lower price, and excellent properties, leading this polymer to be used in many applications [44,45,46]. Regarding the use as a catalyst support material, PDMS offers a great number of possibilities being that the PDMS sponges are most used as the larger the surface, the more nanoparticles adhere, and the more efficient the catalyst will be. PDMS sponges are used as a supporting material for dip-catalysis [47,48,49,50,51], loading the metal NPs by a previous functionalization of the PDMS surface and anchoring pre-synthetized NPs.

Another advantage of some supports of macroscopic dimensions is that they can be used for industrial applications. They have a large surface area but also a hollow structure with a porous wall that is advantageous to facilitating mass transport. These kinds of catalysts achieve maximum recyclability in continuous flow reactions, which are amenable for an automated process. In this sense, Scaiano [52] deposited nanoparticles on glass wool to support the development of heterogeneous catalysts with excellent potential for flow photochemistry applications. Chen [53] was capable of growing vertical gold nanowires on glass fibers to improve both the catalyst loading per unit support area and the flow rate. The commercial availability of polymers makes them good candidates to prepare dip-catalysts [54,55]. Gautam [56] loaded Pd nanoparticles on polyurethane foams with pore-size tunability to catalyze Suzuki coupling reactions. The catalyst showed high stability under reflux conditions and excellent conversion efficiencies with a very minimal loss in catalytic activity for a large number of consecutive catalytic cycles.

In this study, the support chosen to load the Au and Pd NPs was the PDMS since it is a polymer that allows the fabrication of sponges in a simple and fast way using sugar cubes as molds. The 3D porous structure of the molds allows the fabrication of supports that offer a large surface area on which metallic NPs can be deposited through an “in situ” reduction of the metal ion. The catalysts were characterized using scanning electron microscopy (SEM) and energy dispersive X-ray analysis (EDX). Inductively coupled plasma–optical emission spectrometry (ICP-OES) was used to estimate the amount of metallic NPs loaded onto the supports. The advantage of these catalysts manufactured using PDMS is the great variety of geometries that can be obtained. In our case, two simple geometries were chosen, i.e., a cube and a disk. The catalyst as-synthesized showed high stability and facile recovery. The porous structure of the catalyst enables it to present high catalytic activity and good recyclability, both in static conditions and in continuous flow.

The reaction chosen to evaluate the catalytic capacity of the functionalized sponges with metal NPs was the reduction of 4 -*p*-nitrophenol (p-NP) to 4 -*p*-aminophenol employing sodium borohydride. The reaction, of industrial interest [57], can be easily monitored by UV-vis spectroscopy and is widely used to evaluate the catalytic activity of metal nanoparticles in an aqueous solution [58].

## 2. Experimental Part

### 2.1. Chemicals and Materials

Tetrachloroauric acid trihydrate (HAuCl_4_.3H_2_O), potassium tetrachloropalladate (II) (K_2_PdCl_4_), sodium borohydride (NaBH_4_), L-ascorbic acid (>99%), sodium citrate, and p-NP were purchased from Sigma-Aldrich (Madrid, Spain) and used without any further purification. Poly(dymethylsiloxane) (PDMS, Sylgard-184) was purchased from Dow-Corning (Tarragona, Spain), ethanol and sodium hydroxide from Thermo-Fisher (Madrid, Spain), and white sugar from the brand “Azucarera” (Madrid, Spain) was purchased in the supermarket. Milli-Q water was used throughout all the experiments.

### 2.2. PDMS Sponges

To prepare the PDMS, we followed a reported method [59] based on the mixture of 10:1 *w*/*w* (silicone elastomer: curing agent). After mixing into a homogeneous medium, it was centrifuged at 2500 rpm for 2 min to remove the bubbles formed. To obtain a cubic sponge, we introduce a sugar cube inside the PDMS mixture and placed it in the desiccator for 1 min. To obtain the disc sponges, a 10 mL syringe was filled with white sugar then the PDMS mixture was slowly pushed to fill all the syringes with the sugar inside. Both structures were kept in the oven at 60 °C for 3 h. Once the PDMS was cured, both the cubes and the syringe were taken out from the oven. The cubes were washed with several cycles of ethanol and water under sonication to dissolve the sugar. The syringes were demolded to obtain a cylindrical structure of PDMS and sugar. These cylinders were cut into discs of 5 cm in width. These discs were washed similarly to the cubes, with several cycles of ethanol and water under sonication.

### 2.3. PDMS Supported Gold or Palladium Catalyst

Once the support material was done, it was introduced into the UV/Ozone chamber to activate the PDMS surface, making it more hydrophilic. Just after the PDMS activation, different conditions, metal salt, and infiltration times were tested. The PDMS was submerged in 10 mL of metal salt solution (gold or palladium) 10, 25, or 50 mM for 10 or 30 min. After this time, the substrates were removed from the metal solution and submerged immediately in 20 mL of NaBH_4_ (0.05 M) to reduce the metal atoms on the PDMS surface. To eliminate the unattached nanoparticles, the sponges were washed by submerging them again in water.

### 2.4. Reduction of p-NP to p-AP

Before initiating the reaction, the catalyst was placed for 5 min in the UV/Ozone chamber. Meanwhile, in a glass vial, 10 mL of p-NP (10^−4^ M) was mixed with 10 mL of NaBH_4_ (0.2 M) in 0.1 M of NaOH. By adding NaBH_4_ we produced the conversion of p-NP to 4-nitrophenolate ions, a molecule with an absorption peak at 400 nm. To initiate the reaction, the catalysts were immersed after the UV/Ozone into the reaction vial. The reaction progress was monitored by withdrawing an aliquot at a designated time and measuring the absorbance with an UV-visible-NIR spectrophotometer (Agilent, Madrid, Spain) using a 1 cm path length quartz cuvette and placing it back into the reaction medium instantaneously after the absorbance measurement. As the reaction progressed, the absorption peak at 400 nm, which corresponds to 4-nitrophenolate ions, decreased gradually and the peak at 300 nm corresponding to 4-AP increased.

### 2.5. Catalyst Recycling

The catalyst was recycled simply by removing it from the reaction medium, then washing it with water, and drying it at room temperature. In each catalytic reaction, the catalyst was introduced into the UV/Ozone chamber for 5 min to activate the surface.

### 2.6. Continuous Flow Reaction

The catalyst was activated by the UV/Ozone treatment and it was introduced in a 10 mL syringe. Then, the reaction mixture was introduced in the syringe and it was placed on the injection pump. Once the syringe was empty, more reaction mixture was introduced again in the same syringe with the same catalyst. This process was repeated 8 times.

### 2.7. Characterization

UV/Ozone ProcleanerTM Plus (Madrid, Spain) was used to functionalize the PDMS surfaces. A spectrophotometer UV-Vis Agilent Technologies Cary 8454 (Santa Clara, CA, USA) was used to follow the kinetic reactions. SEM images were obtained using a FEG JEOL JSM 6700F (Tokyo, Japan). For the flow reaction, an injection pump Harvard apparatus PHD 2000 (Holliston, MA, USA) was used. ICP, Perkin Elmer Optima 4300 DV (Markham, ON, Canada), was used to determine the amount of metal salt (gold or palladium) reduced on the PDMS sponges.

## 3. Results and Discussion

The supported catalyst was performed following a reported method [59] based on the mixture of silicone elastomer and curing agent (see a detailed in the Section 2). PDMS rectangular prisms with average dimensions 1.7 cm length, 1.7 cm width, and 1.2 cm height were initially created to test the reaction in static conditions. By fully covering a sugar cube with the polymer mixture, it was possible to obtain the negative of the sugar cube with PDMS, as shown in Figure S1.

**Scheme 1:**
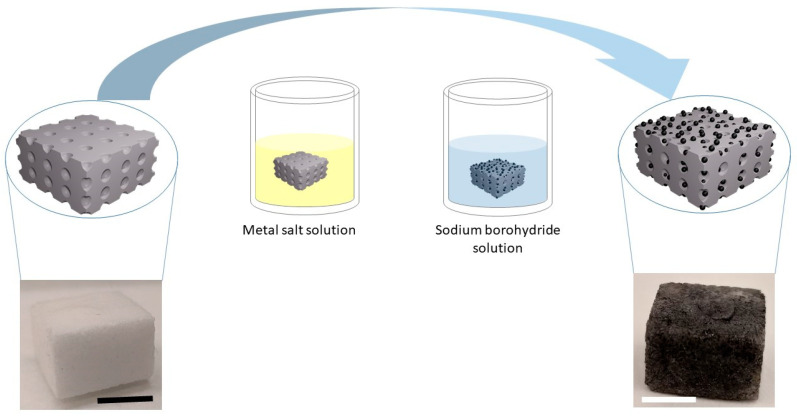
Schematic representation of the synthesis process for the catalysts based on PDMS sponges supported metal nanoparticles. Scale bars represent 1 cm.

Once the PDMS rectangular prisms were obtained, they were washed with water and ethanol to remove the sugar. Once the sugar was removed, they were introduced into an aqueous salt solution to study the influence of the infiltration time and the salt concentration on the process (Scheme 1). To discern the best synthetic conditions in terms of nanoparticle loading, two different infiltration times (10 and 30 min) and three different Au or Pd salt concentrations (10, 25, and 50 mM) were tested. The rectangular prisms were immersed inside the metal salt solutions and right after the selected time they were dipped in a borohydride solution to reduce the metal ions and obtain the final nanoparticles. Once the nanoparticles were synthesized on the porous surface of the PDMS rectangular prisms, the amount of reduced metal was determined by acid digestion (using aqua-regia) and characterized by inductively coupled plasma-optical emission spectrometry (ICP-OES). As can be seen in Figure S2A,B, the infiltration time is not relevant, since after 10 min the same gold or palladium atoms were loaded as after 30 min. On the contrary, the concentration of salt in which the PDMS rectangular prisms were immersed had a very notorious influence on the amount of metal loaded on its surface. Then, from the ICP-OES characterization, it can be determined that the higher the salt concentration, the higher amount of gold or palladium will be loaded on the PDMS, and therefore the higher number of active sites the catalyst will have. For this reason, we can consider that the best conditions for the synthesis of the PDMS-supported catalyst are 50 mM metal salt solution for 10 min, and from now on, all catalysts will be made with these characteristics.

**Figure 1:**
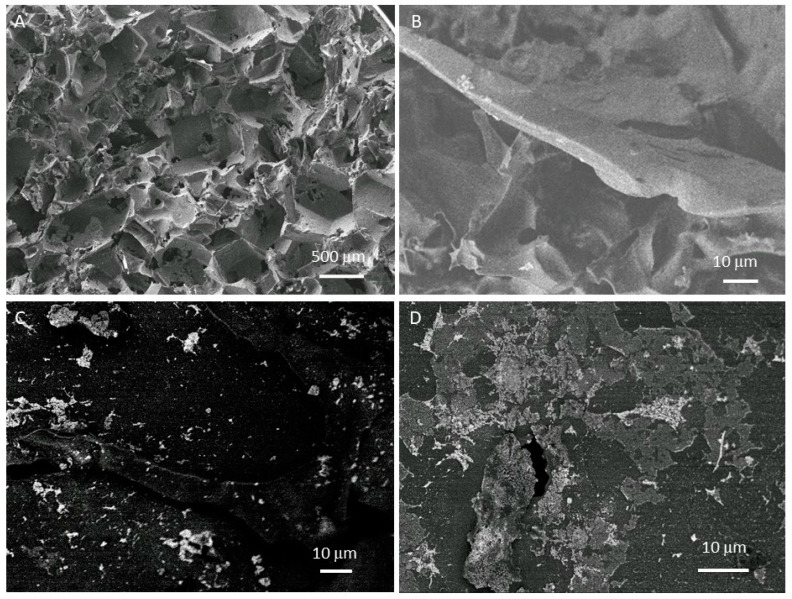
Representative SEM micrographs of PDMS sponges doped with gold nanoparticles. (**A**,**B**) Secondary electron analysis shows the morphology and high porosity of the PDMS sponges. (**C**,**D**) Backscattered electron analysis shows the presence of gold nanoparticles as bright spots.

Furthermore, the rectangular prisms were characterized by scanning electron microscopy (SEM). Thanks to this technique, the porous structure of PDMS can be easily discerned by secondary electron analysis (see Figure 1A,B). A detailed analysis of several SEM images allowed us to estimate an average dimension of the pores of 291.6 ± 92.3 µm (see Figure S3 in the Supplementary Materials). Additionally, Figure 1C,D shows the backscattered electron analysis in which the presence of metal nanoparticles can be identified as bright spots on the surface of the PDMS. It has to be noted that the SEM characterization was complex, as the PDMS is not a conductive polymer and the samples were charged during the characterization. Nevertheless, it can be seen how the nanoparticles followed a random distribution throughout the surface of the PDMS rectangular prisms. Moreover, as is shown in Figure S4 in the Supplementary Materials, the Energy Dispersive X-ray (EDX) analysis revealed the presence of Si and O, corresponding with the main elements of the PDMS, as well as the presence of Au on the surface of the sponge. Additionally, a detailed analysis allowed us to estimate an average size diameter of 46.6 ± 11.2 nm assuming a spherical morphology (see Figure S5 in the Supplementary Materials).

**Figure 2:**
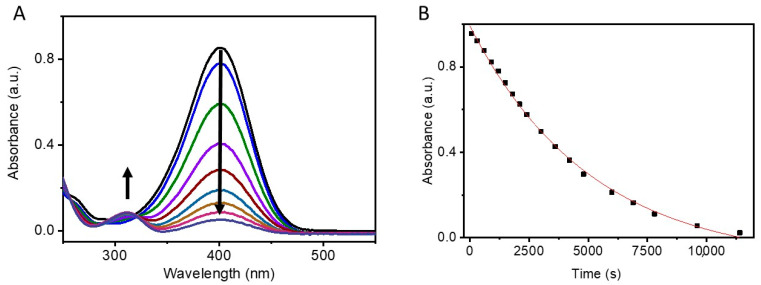
(**A**) Spectral evolution of a mixture of p-NP and borohydride upon addition of one PDMS sponge doped with Au nanoparticles. [p-NP] = 5 × 10^−5^ M, [NaBH_4_] = 0.1 M at pH = 13. (**B**) Kinetic trace of the absorbance at 400 nm during the reduction of p-NP corresponding to (**A**). The solid line shows the best fit of Equation (1) to experimental data, k_app_ = 1.9 × 10^−4^ s^−1^.

To study the performance of our heterogeneous catalyst, we used the reduction of p-NP to *p*-aminophenol by borohydride ions, which is considered a model reaction to test the catalytic activity of metal nanoparticles in an aqueous solution because it is a clean reaction that does not occur in the absence of a catalyst (Figure S6) and it can be easily monitored by UV-vis spectroscopy [58]. As we can see in Figure 2A, the absorption band at 400 nm, due to *p*-nitrophenolate ions, decreased gradually with time, whereas a second peak at 305 nm increased due to the formation of *p*-aminophenol. The isosbestic point at 314 nm indicates that only this product was formed during the reaction. In presence of metal nanoparticles, borohydride transfers hydrogen to the metallic surface. The p-NP molecules, also adsorbed on the surface of the nanoparticles, were reduced to *p*-aminophenol by the surface-hydrogen species in the rate-limiting step of the process [60].

It should also be pointed out that the reaction in the absence of nanoparticles can be neglected because it is very slow as compared with the catalyzed reaction (see Figure S6). Additionally, it is worth mentioning that the hydrolysis of the borohydride ions also occurs and thus competes with the p-NP reduction. Fortunately, this chemical decomposition of the reducing agent can be strongly inhibited by working at a high pH [61]. For this reason, the pH was maintained at 13 for all of the experiments reported here and we can therefore neglect this side reaction. Besides, borohydride concentration is always in large excess compared with p-NP concentration. Thus, the kinetics of the reduction process can be treated as a pseudo-first-order reaction following the equation

(1) $$-\frac{d\left[p-\mathrm{NP}\right]}{\mathrm{dt}}=k_{\mathrm{app}}\left[p-\mathrm{NP}\right]=k_{s}S\left[p-\mathrm{NP}\right]$$

where k_app_ is the apparent rate constant, S is the total surface area available, and k_S_ is the rate constant normalized to the total metallic surface [60]. As we can see in Figure 2B, the decrease in absorbance at 400 nm can be fitted to an exponential first-order decay that lets us obtain the values of k_app_. Recently, Maschmeyer [62] pointed out that the reaction will proceed in a pseudo-first-order regime if [NaBH_4_] is in a large excess over p-NP (more than 200-fold excess), which happened in the present case. If this condition is not fulfilled, the reaction will appear to be zeroth-order in [p-NP]. A constant value of 1.9 × 10^−4^ s^−1^ was obtained when one PDMS sponge loaded with gold nanoparticles was used to catalyze the reaction. 

**Figure 3:**
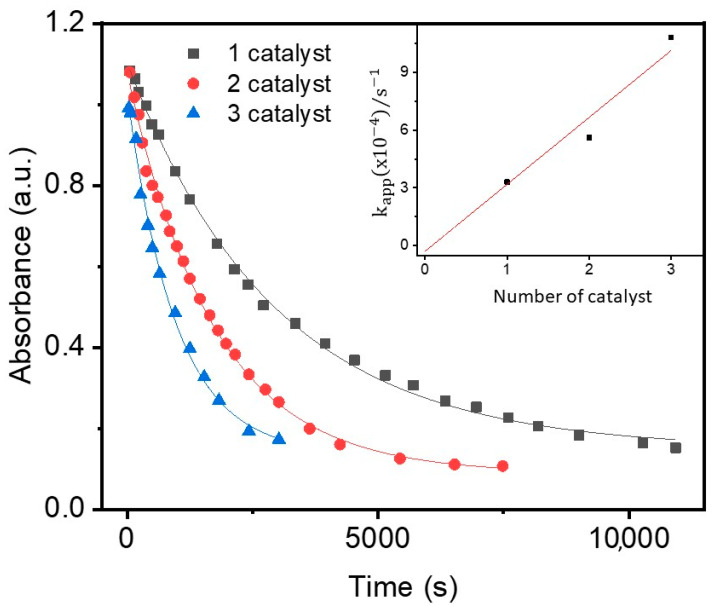
Kinetic trace of the absorbance at 400 nm during the reduction of p-NP in the presence of 1, 2, and 3 PDMS sponges doped with Pd nanoparticles, [p-NP] = 5 × 10^−5^ M, [NaBH_4_] = 0.1 M and pH = 13. The solid lines represent the best fit of Equation (1) to the experimental data, k_app_ = 3.3 × 10^−4^ s^−1^, 5.6 × 10^−4^ s^−1^ and 10.8 × 10^−4^ s^−1^, respectively. The inset shows the variation of k_app_ with the number of Au-doped PDMS sponges. The line represents the best fit of a straight line equation to the experimental data.

To check the catalytic behavior of the supported metal nanoparticles, we studied the influence of catalyst amount on the reaction rate. The experiments were carried out with 1, 2, and 3 catalytic PDMS sponges loaded with metal nanoparticles, keeping constant the amount of p-NP (5 × 10^−5^ M), the concentration of borohydride (0.1 M), and at pH = 13. In Figure 3, it can be seen that in the three cases a good fit to Equation (1) was obtained, which shows that in the presence of Pd nanoparticles as a heterogeneous catalyst, the reactions follow a first-order kinetic. Similar results were obtained when gold nanoparticles were used (see Figure S7 in Supplementary Materials). Table 1 summarizes the kinetic parameter obtained. As expected, the apparent rate constants followed a linear trend with the number of PDMS sponges employed because the increase in surface area is proportional to the number of catalytic sponges.

As we mentioned in the introduction section, small colloidal metal nanoparticles tend to aggregate and coalesce together. To avoid this aggregation, we used a polymeric sponge as a supporting material. In the present case, considering that the nanoparticle synthesis was performed in situ, they presented a broad size distribution and because of the fact that the particles are supported on the PDMS substrate, rendering its surface partially useless, an accurate estimation of the available catalytic surface is not possible. Therefore, to evaluate and compare the catalytic efficiency of these heterogeneous catalysts, we calculated the turnover frequency (TOF), a semi-quantitative parameter widely used in heterogeneous catalysis that quantifies the specific capacity of a catalytic center for a reaction under defined conditions.

(2) $$\mathrm{TOF}=\frac{\mathrm{mols}\;\mathrm{of}\;\mathrm{obtained}\;\mathrm{product}}{\mathrm{mols}\;\mathrm{of}\;\mathrm{catalyst}\;\times \;t}\;$$

where t is the time required for a reaction to reach 80% completion. In this work, in order to be able to compare all the studies, the TOFs were calculated taking into account the total amount of metallic NPs loaded into the catalyst support, obtained from ICP measurements, not the available surface. 

Table 1 summarizes the apparent rate constants and the calculated TOFs under the same experimental conditions for Au-doped and Pd-doped PDMS sponges. Pd supported nanoparticles offer a higher catalytic activity (mean TOF values of 1.10 × 10^−4^ s^−1^) than those of gold (mean TOF values of 6.4 × 10^−5^ s^−1^). A similar trend was recently reported by Schlücker and col [51] for unsupported nanoparticles.

Opposite to the rate constant values, the values of the TOF numbers kept almost constant with the number of sponges for both metallic nanoparticles. This is a logical result because, as we can see in Equation (2), the definition TOF number is already taking into account the amount of catalyst used and indicating to us that the surface area and the active sites are almost the same in each catalytic sponge.

To compare the TOF obtained in this study with others using different heterogeneous supports, we have to keep in mind how the experimental conditions affect our model reaction. Ballauf and co-workers [25,49,50] extensively studied this model reaction and they found that the reaction rate increases with the concentration of borohydride until leveling off. The pH also has an important effect on the reaction rate and mechanism [61]. In the case of palladium as a catalyst, to the best of our knowledge, the higher TOF value (5.2 s^−1^) was reported by Bai [63] for Pd NPs anchored on amine-functionalized silica nanotubes, but working at very high [NaBH_4_] = 1.76 M, which increases the reaction rate. Using a lower borohydride concentration of 0.06 M, a high TOF value of 0.18 s^−1^ was reported by Gautam [56] for Pd nanoparticles loaded on polyurethane foams. In the case of gold as a catalyst, Chen [53] estimated a TOF value of 0.09 s^−1^ for Au nanowires supported on glass fibers, but using 2.5 times higher borohydride concentration than in the present case, leading to smaller reaction times and then higher TOFs. Lower TOF (0.03 s^−1^) has been reported for 3–5 nm Au NPs anchored on hollow microspheres [64] and a value of 0.02 s^−1^ has been found for 15 nm Au NPs embedded in porous silica shells [65]. Smaller gold nanoparticles (2–3 nm) stabilized by ionic polymers [66] show a TOF value of 0.07 s^−1^. Unfortunately, attempts to compare literature data are difficult due to unreported concentrations and experimental conditions, which is a practice recently highlighted by Baker and coworkers [67].

**Figure 4:**
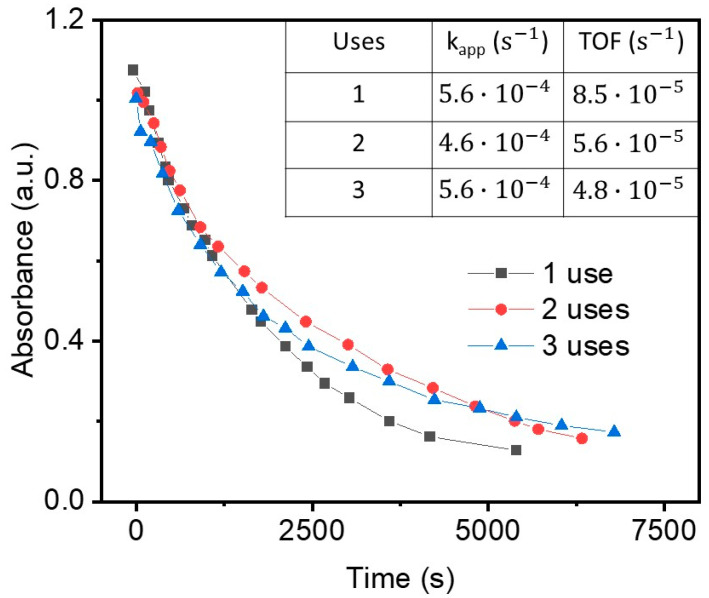
Kinetic trace of the absorbance at 400 nm during the reduction of p-NP in the presence of the same PDMS sponge doped with Pd NPs for different recycle numbers, as indicated. [p-NP] = 5 × 10^−5^ M, [NaBH_4_] = 0.1 M and pH = 13. The solid lines show the best fit of Equation (1) to experimental data. The inset shows the k_app_ and the TOFs values calculated to each use.

An important advantage of heterogeneous dip-catalysts over homogeneous ones is that they can be easily recovered and reused. To check the recyclability of our catalysts, three kinetic tests were carried out, always using the same catalyst sponges. After each use, the sponges were recovered from the solution, washed with water and ethanol, and reused. As we can see in Figure 4 for Pd NPs, the catalysts not only were capable to transform the 100% of reactant into the product in each use, but also did so with almost no loss in catalytic efficiency, the values of the rate constant and TOF keeping constant until the third use. Similar results were obtained when gold nanoparticles were supported on the PDMS sponges (see Figure S8 in the Supplementary Materials).

**Figure 5:**
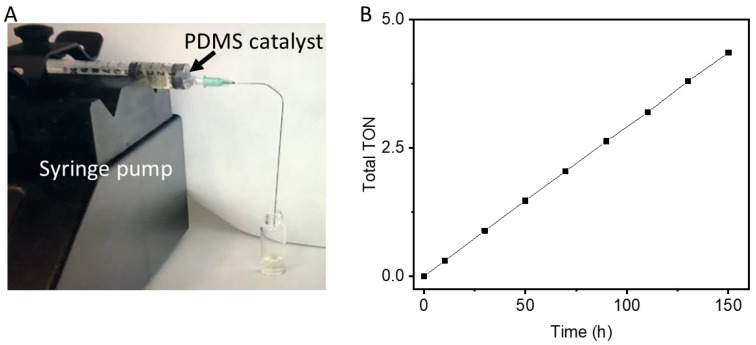
(**A**) Photograph showing the experimental set-up to study the continuous flow reaction. The arrow indicates the Pd-doped PDMS disc located at the end of the syringe. (**B**) Representation of the total TON versus time for the continuous flow reaction for one Pd-doped PMDS as catalyst.

Once the catalytic behavior of the PDMS sponges was determined, we decided to study the performance of the supported metallic NPs in a continuous-flow reaction. We think this is a better way to study the catalytic and the long-lasting activity of the catalyst because it avoids the recycling step, which is time-consuming and expensive, and besides, can be considered as an approximation to industrial conditions. For that, we placed inside a 10 mL syringe a PDMS disc of the same diameter of the disc (1.4 cm) and 1 cm in height (see Figure S1 in the Supplementary Materials). On the other hand, 5 mL of 1 × 10^−4^ M of p-NP was mixed with 5 mL solution of 0.2 M of ${\mathrm{BH}}_{4}^{-}$ and the mixture was forced to pass through the catalytic disc. In order to maintain a constant flow, we have used a pressure pump (see Figure 5A). Considering the mean values for the TOF calculated, from the studies with 1, 2, or 3 catalytic discs (1.10 × 10^−4^ s^−1^ for Pd NPs and 6.4 × 10^−5^ s^−1^ for Au NPs), the obtained ICP-OES data and due to the fact that the catalytic discs are 1 cm in length, it was possible to calculate that, for a total conversion of the p-PN into products, the flow rate value should not be higher than 1.0 mL/h. Therefore, we decided to set a flow rate of 0.5 mL/h. To control the capacity and recyclability of the catalyst, the resulting solution was collected in vials and the degree of conversion into products was obtained measuring the decrease in absorbance at 400 nm. Once all the volume passed through the catalyst, the syringe was refilled with fresh solution and the process was repeated. After each cycle, we calculated the Turnover Number (TON) parameter (see Equation (3)), which indicates the amount of reactant that can be transformed into products by the catalyst without any loss of efficiency. As is shown in Figure 5B, the TON of Pd catalyst did not decrease after 160 h (or 80 mL of reactants), demonstrating that the catalyst continues acting without any loss of catalytic efficiency. Similar results were obtained when Au was used, but in this case, the process was followed for 45 h (see Figure S9 in the Supplementary Materials).

(3) $$\mathrm{TON}=\frac{\mathrm{mol}\;\mathrm{of}\;\mathrm{product}\;\mathrm{obtained}}{\mathrm{mol}\;\mathrm{of}\;\mathrm{catalyst}}\;$$

## 4. Conclusions

In summary, we reported a novel approach to synthesize a dip catalyst based on polydimethylsiloxane sponge-supported metal nanoparticles. The proposed approach was successfully developed for Au and Pd. Both metals, gold and palladium, showed excellent recyclability under static reaction conditions towards a model reaction such as the hydrogenation of p-NP. Additionally, the dip-catalyst was used for continuous flow reactions, observing that the efficiency was still the maximum even after 150 h of use, opening a new way of applying catalyst supported PDMS sponges in the chemical industry.

## Figures and Tables

**Table 1:** Apparent rate constants and the calculated TOFs, under the same experimental conditions ([p-NP] = 5 × 10^−5^ M, [NaBH_4_] = 0.1 M and pH = 13) for Au-doped and Pd-doped PDMS sponges.

	Gold		Palladium	
	k_app_/s^−1^	TOF/s^−1^	k_app_/s^−1^	TOF/s^−1^
1 sponge	1.9 × 10^−4^	7.0 × 10^−5^	3.3 × 10^−4^	1.03 × 10^−4^
2 sponge	3.8 × 10^−4^	6.1 × 10^−5^	5.6 × 10^−4^	0.85 × 10^−4^
3 sponge	6.1 × 10^−4^	6.0 × 10^−5^	10.8 × 10^−4^	1.10 × 10^−4^

## Supplementary Materials

The following supporting information can be downloaded at: https://www.mdpi.com/article/10.3390/nano12122081/s1, Figure S1. Photograph of a PDMS sponge with a shape of a rectangular prism (A) and a disc (C) before and after ((B) and (D)) the “in situ” synthesis of Pd nanoparticles. Scale bar or 1 cm; Figure S2. ICP-OES data for different times of infiltration and different metal concentrations for gold (A) and palladium (B). Experiments done in triplicate; Figure S3. SEM micrograph (A) of the PDMS sponges and the histogram of the pores sizes (B); Figure S4. SEM micrographs (A and B) and EDX (C) of the gold nanoparticles supported PDMS sponges; Figure S5. SEM micrographs of the nanoparticles synthesized on the PDMS sponges (A) and the histogram (B) of the nanoparticles diameter; Figure S6. Representation of the absorbance at 400 nm vs time for the reaction in the absence of catalyst; Figure S7. (A) Representation of the absorbance at 400 nm versus time for the reactions with 1, 2 and 3 Au loaded PDMS sponges. (Inset) Table summarizing the rate constant obtained and the calculated TOF for each experiment. (B) Variation of the rate constant values versus the number of Au loaded PDMS sponges. The line represents a linear fit to the data; Figure S8. Kinetic trace of the absorbance at 400 nm during the reduction of p-NP in the presence of the same PDMS sponge doped with Au nanoparticles for different recycle numbers, as indicated. [p-NP] = 5 × $\;10^{-5}$ M, [NaBH_4_] = 0.1 M and pH = 13. The solid lines show the best fit of Equation (1) to experimental data; Figure S9. Representation of the total TON versus time for the continuous flow reaction for Au-doped PMDS as catalyst.
