# Supplementary material for: Polydimethylsiloxane Sponge-Supported Metal Nanoparticles as Reusable Catalyst for Continuous Flow Reactions
Source: Nanomaterials (Basel). 2022 Jun 16;12(12):2081. doi: 10.3390/nano12122081 (PMC9227176; doi:10.3390/nano12122081)
Supplement: Supplementary file 1 [file nanomaterials-12-02081-s001.zip › nanomaterials-1757470-supplementary.pdf]

nanomaterials

MDPI

Supplementary Material

Polydimethylsiloxane Sponge-Supported Metal Nanoparticles as Reusable Catalyst for Continuous Flow Reactions

Sergio Gómez-Graña [1,2,*], Marta Pita [1,2], Paula Humada-Iglesias [1,2], Jorge Pérez-Juste [1,2] and Pablo Hervés [1,2,*]

1 CINBIO, Departamento de Química Física, Universidade de Vigo, 36310 Vigo, Spain; pitfermarta@gmail.com (M.P.-F.); humadapaula@gmail.com (P.H.-I.); juste@uvigo.es (J.P.-J.)
2 Instituto de Investigación Sanitaria Galicia Sur, Hospital Álvaro Cunqueiro, 36213 Vigo, Spain
* Correspondence: segomez@uvigo.es (S.G.-G.); jherves@uvigo.es (P.H.)

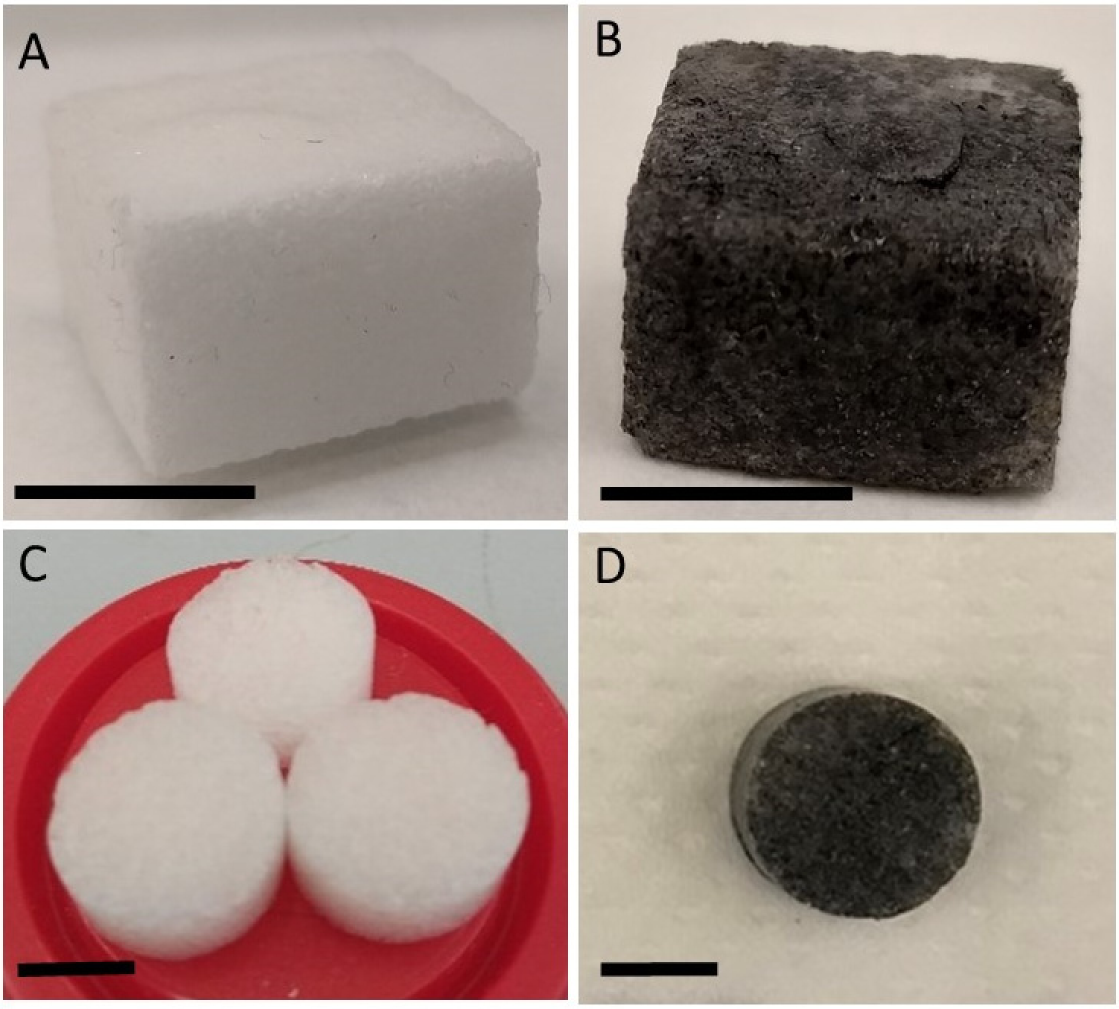

Figure S1. Photograph of a PDMS sponge with a shape of a rectangular prism (**A**) and a disc (**C**) before and after ((**B**) and (**D**)) the "in situ" synthesis of Pd nanoparticles. Scale bar or 1 cm.

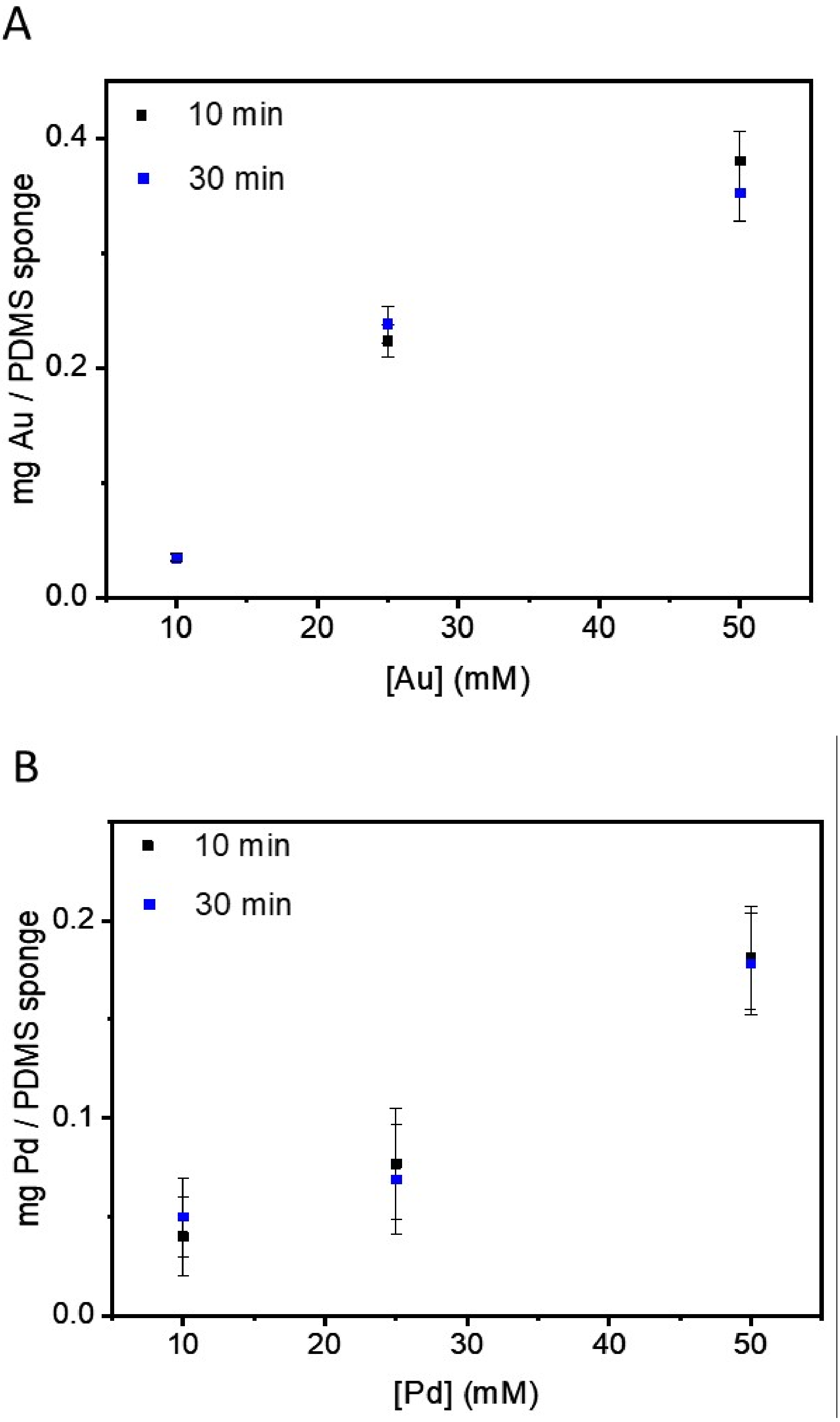

Figure S2. ICP-OES data for different times of infiltration and different metal concentrations for gold (**A**) and palladium (**B**). Experiments done in triplicate.

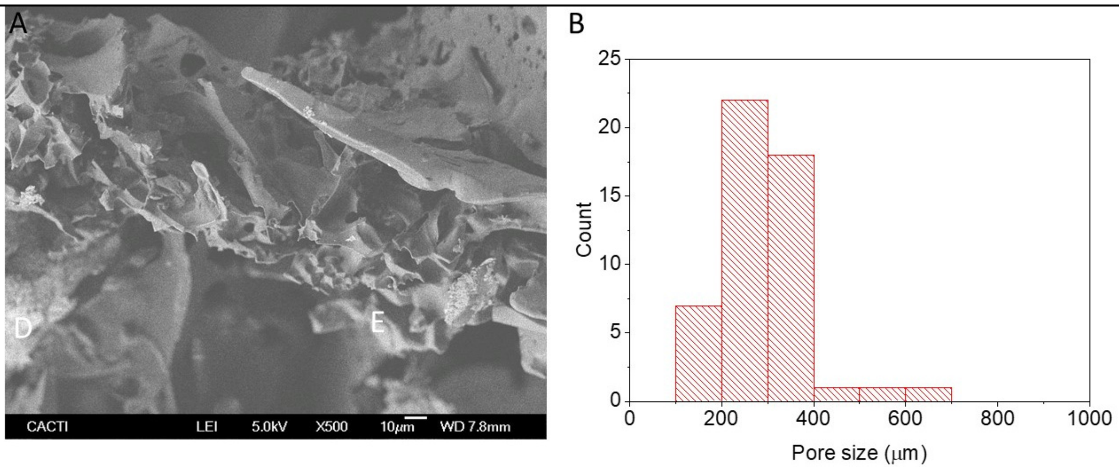

Figure S3. SEM micrograph (**A**) of the PDMS sponges and the histogram of the pores sizes (**B**).

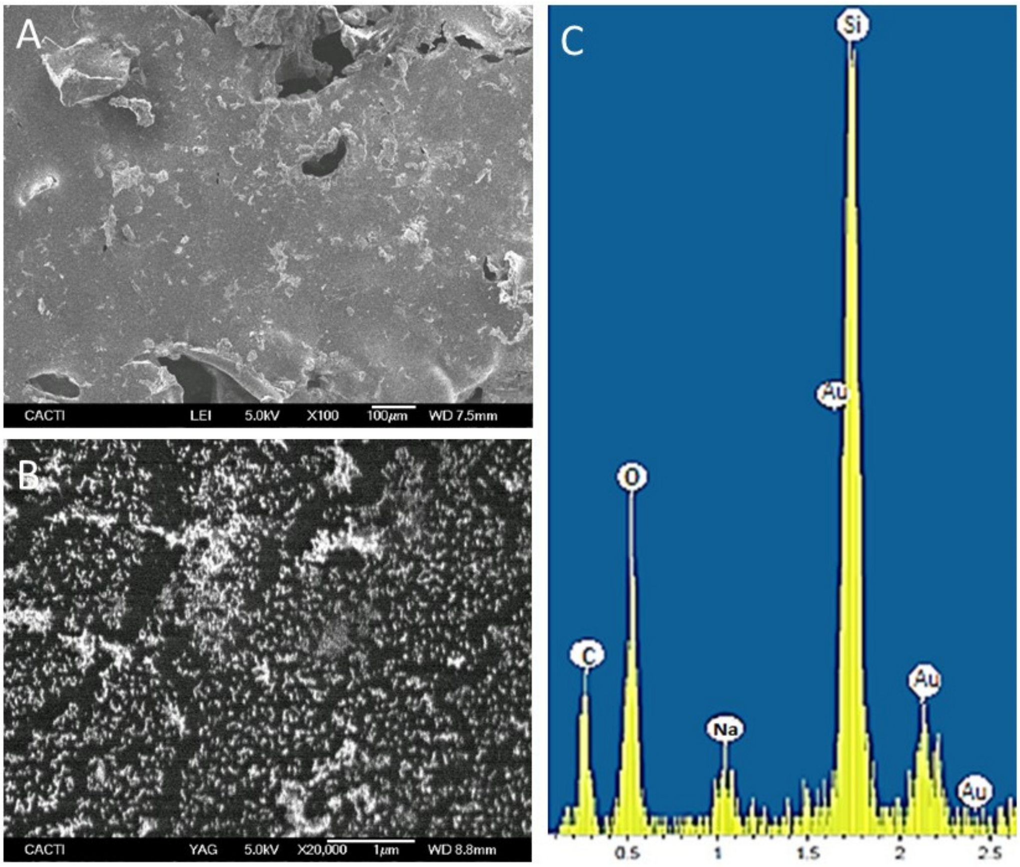

Figure S4. SEM micrographs (**A** and **B**) and EDX (**C**) of the gold nanoparticles supported PDMS sponges.

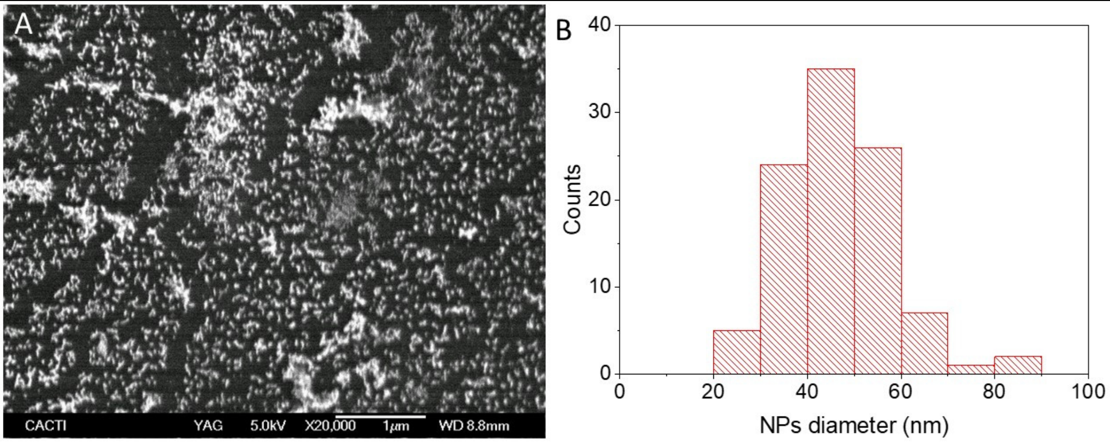

Figure S5. SEM micrographs of the nanoparticles synthesized on the PDMS sponges (**A**) and the histogram (**B**) of the nanoparticles diameter.

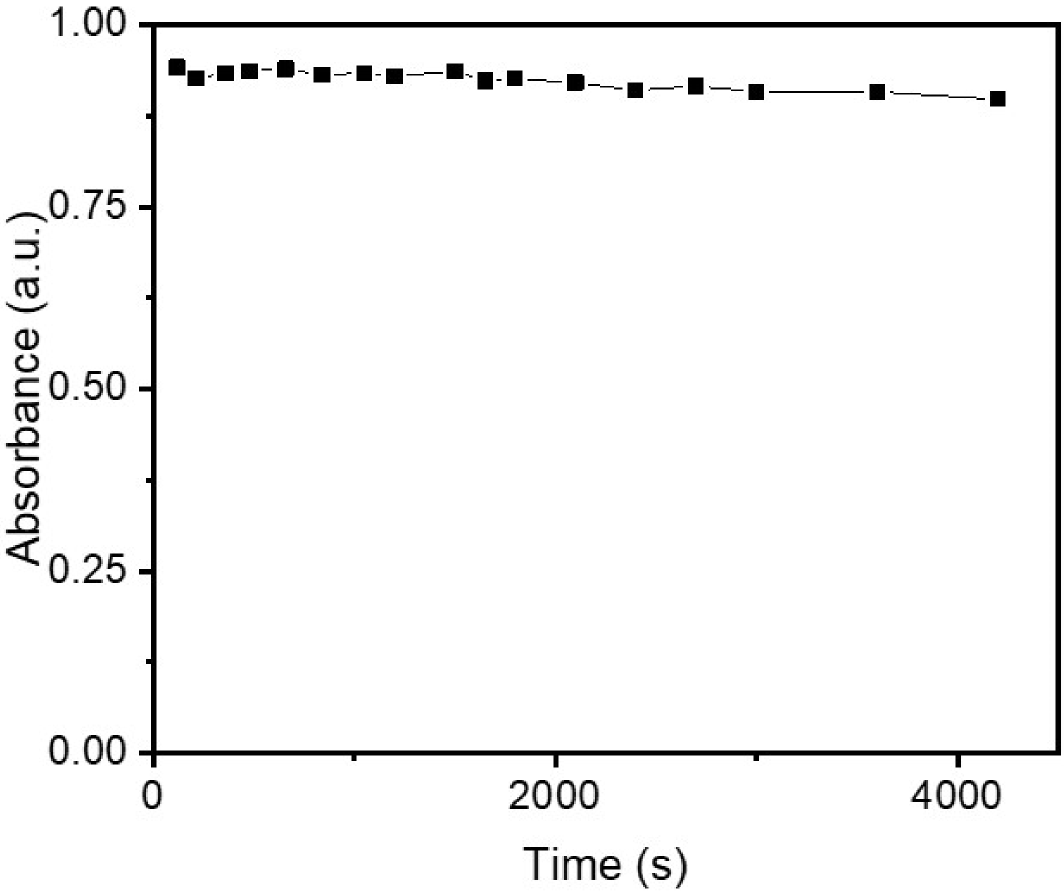

Figure S6. Representation of the absorbance at 400 nm vs time for the reaction in the absence of catalyst.

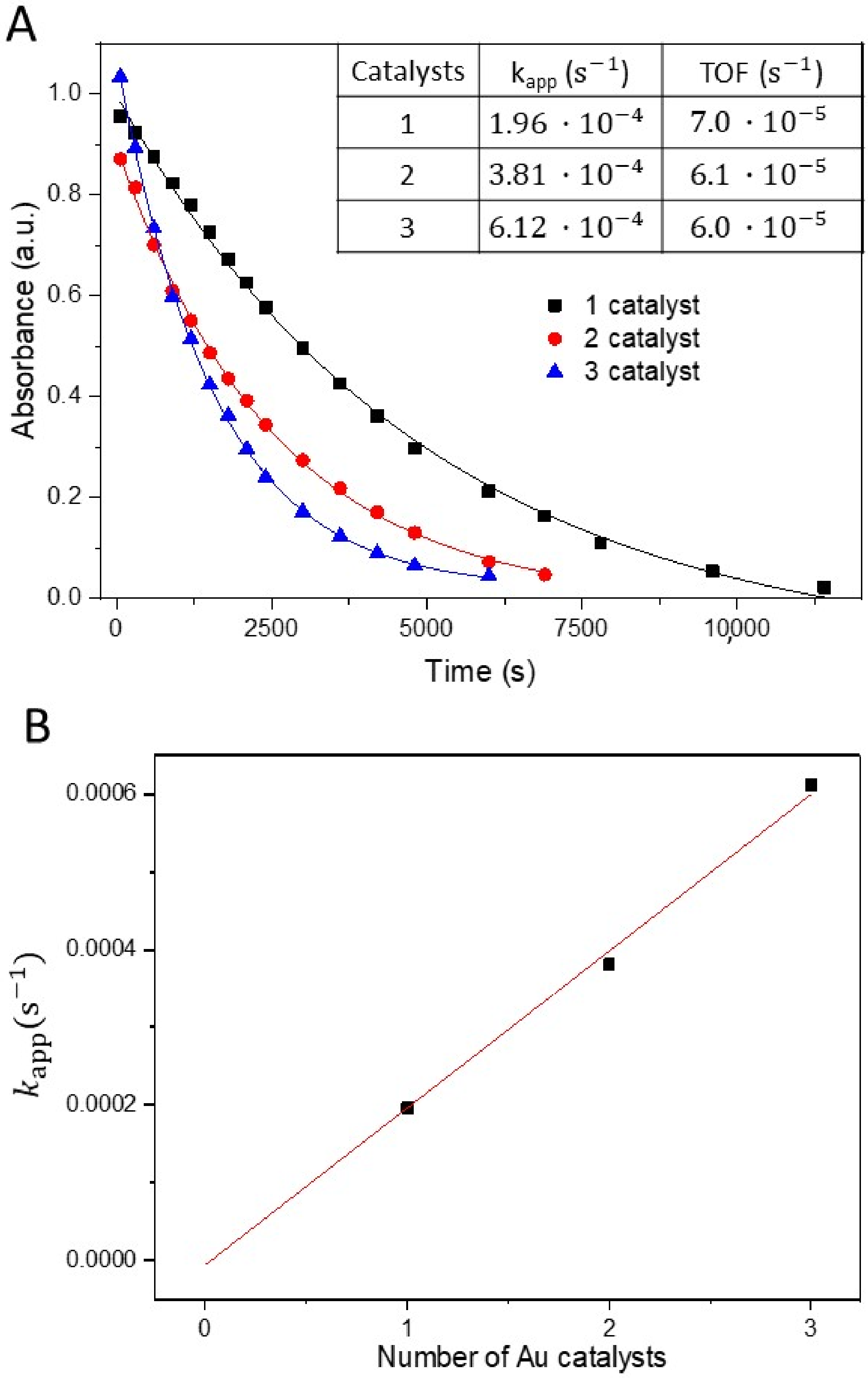

Figure S7. (**A**) Representation of the absorbance at 400 nm versus time for the reactions with 1, 2 and 3 Au loaded PDMS sponges. (Inset) Table summarizing the rate constant obtained and the calculated TOF for each experiment. (**B**) Variation of the rate constant values versus the number of Au loaded PDMS sponges. The line represents a linear fit to the data.

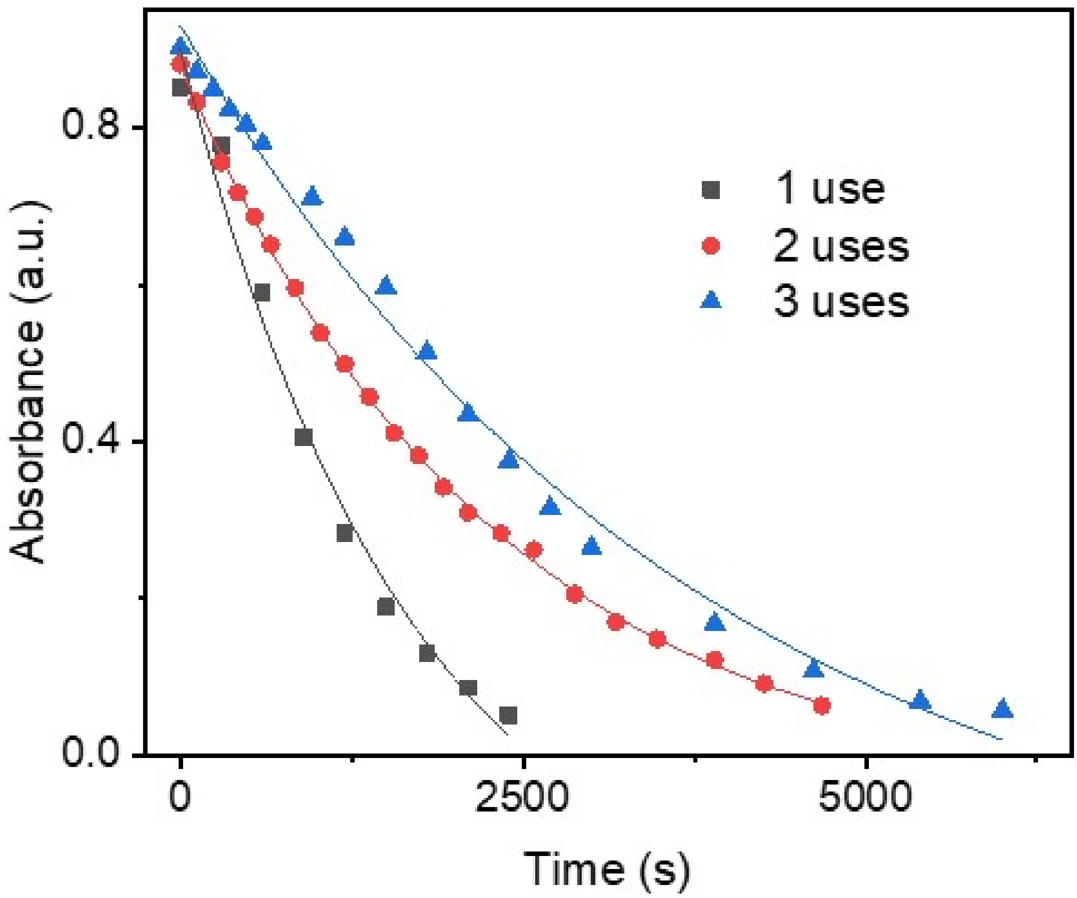

Figure S8. Kinetic trace of the absorbance at 400 nm during the reduction of p-NP in the presence of the same PDMS sponge doped with Au nanoparticles for different recycle numbers, as indicated. [p-NP]= 5 x 10^{-5} M, [$NaBH_4$]= 0.1 M and pH= 13. The solid lines show the best fit of Equation 1 to experimental data.

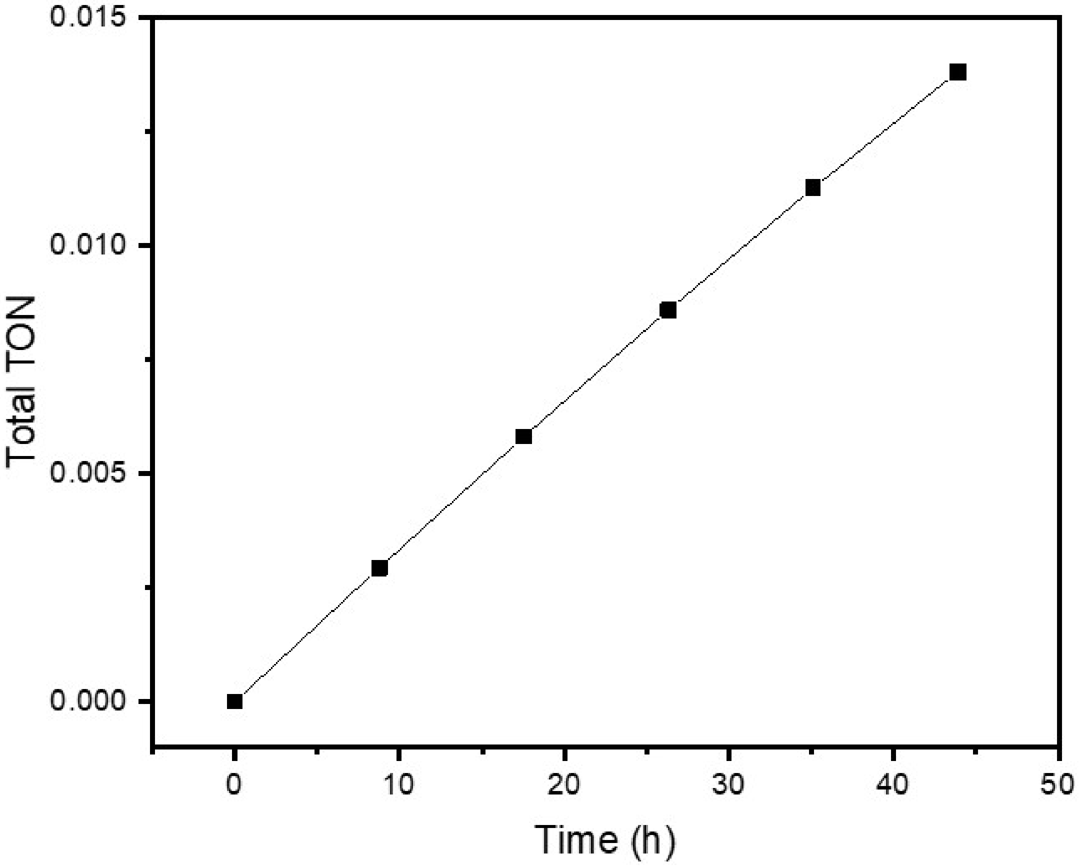

Figure S9. Representation of the total TON versus time for the continuous flow reaction for Au-doped PMDS as catalyst.
